# Effect of Glass Fibers Thermal Treatment on the Mechanical and Thermal Behavior of Polysulfone Based Composites

**DOI:** 10.3390/polym12040902

**Published:** 2020-04-13

**Authors:** Galal Sherif, Dilyus I. Chukov, Victor V. Tcherdyntsev, Valerii G. Torokhov, Dmitry D. Zherebtsov

**Affiliations:** 1 Center of composite materials, National University of Science and Technology “MISIS”, Leninskiy prosp. 4, Moscow 119049, Russia; dil_chukov@mail.ru (D.I.C.); vvch@misis.ru (V.V.T.); vgtorohov@gmail.com (V.G.T.); dmitry_zherebtsov@bk.ru (D.D.Z.); 2 Production and Design Dept., Faculty of Engineering, Minia University, Minia 61111, Egypt

**Keywords:** glass fibers, heat treatment, polysulfone, mechanical properties

## Abstract

The effect of thermal treatment of glass fibers (GF) on the mechanical and thermo-mechanical properties of polysulfone (PSU) based composites reinforced with GF was investigated. Flexural and shear tests were used to study the composites’ mechanical properties. A dynamic mechanical analysis (DMA) and a heat deflection temperature (HDT) test were used to study the thermo-mechanical properties of composites. The chemical structure of the composites was studied using IR-spectroscopy, and scanning electron microscopy (SEM) was used to illustrate the microstructure of the fracture surface. Three fiber to polymer ratios of initial and preheated GF composites (50/50, 60/40, 70/30 (wt.%)) were studied. The results showed that the mechanical and thermo-mechanical properties improved with an increase in the fiber to polymer ratio. The interfacial adhesion in the preheated composites enhanced as a result of removing the sizing coating during the thermal treatment of GF, which improved the properties of the preheated composites compared with the composites reinforced with initial untreated fibers. The SEM images showed a good distribution of the polymer on the GF surface in the preheated GF composites.

## 1. Introduction

Polysulfone (PSU) is a high-performance amorphous thermoplastic with excellent mechanical properties, high service temperature due to its high glass transition temperature (*T*_g_) 185 °C, flexibility, and excellent thermal stability. These superior properties make PSU the most appropriate choice for wide applications such as medicine, food, processing equipment, and relatively high-temperature components [1,2,3,4]. PSU is broadly used because of increasing demands for high-temperature polymers in many industries, such as automotive, aerospace, and microelectronics. 

Many materials and types of fillers are used to reinforce polymer matrix composites [5,6,7,8]. Nowadays, most of the high-performance polymer-based composite materials are produced using fibrous fillers [4,5,6,7,8,9]. GF are one of these materials that offer high specific strength and stiffness, low cost, and suitable heat resistance [6,10,11]. The mechanical properties of composites mainly depend on reinforcing fiber/matrix properties, fibers’ surface morphology, and the interfacial bonding between the fiber and the matrix [8,10]. It is also recognized that the bonding strength at the fiber–matrix interface has a significant effect on composite materials’ mechanical properties. Therefore, so far, many efforts have been made to propose an appropriate engineered fiber/matrix interface to significantly increase the composite’s strength, toughness, and environmental stability [11,12]. As it is known, the bonding strength mainly depends on physical absorption, chemical reaction, and bonding between the fiber surface layer and the matrix polymer. The bonding strength is strongly affected by a fiber surface modification such as surface treatment or chemical sizing [10]. 

Several studies dealing with thermoplastic composites reveal that temperature has a noticeable influence on mechanical properties [12,13,14,15,16,17,18]. It has been found that tensile strength and Young’s modulus of thermoplastic composites decrease with increasing temperature and drop sharply close to the *T*_g_ [19,20]. On the other hand, above the *T*_g_, the strain increases because of the intensive motion of polymers’ molecular chains. This effect in unfilled polymers is greater than that of in reinforced polymers [21,22]. The effect of temperature and different environmental conditions on the thermoplastic reinforced with GF was studied, and it was observed that a bi-linear reduction in strength and stiffness occurred in the *T*_g_ range [23,24]. 

In addition to traditional methods of polymer composites mechanical properties investigations, such as tensile, compression, flexural tests, etc., thermal and dynamical tests methods are widely used nowadays. One of these methods is dynamic mechanical analysis (DMA), which is widely and successfully used to study the dynamic mechanical response of composites. The data used as a function of temperature, time, frequency, and stress can also be an indicator of the interface, morphology, and presence of an internal defect in the composite structure. It is an excellent technique to study the effect of temperature on the mechanical properties of composite materials. Since polymeric composites in many applications exposed to different types of dynamic stressing during service, studying the viscoelastic behavior of these materials have become critical [25,26,27]. The heat deflection temperature (HDT) test is another effective tool to evaluate the physical performance of a polymer under load and elevated temperature. The HDT data represent the maximum service temperature without a large deflection [28,29,30,31].

Recently [32], we investigated the effect of the formation route on the GF reinforced polyethersulfone based composites. It was observed that composite formation via compression molding of a polymer powder together with GF does not allow samples to be obtained with high mechanical properties, whereas the formation of composites by GF impregnation with polyethersulfone solution results in the formation of composites with high flexural strength. Therefore, in the present study, we applied the polymer solution route to obtain PSU based composites. The current study aims to illustrate the thermal treatment effect of the removal of GF sizing coating on the mechanical and thermal properties of PSU composites. According to our results, the mechanical and thermal properties increase with an addition of a preheated GF, which leads to an expansion of high-temperature applications of these composites. Additionally, the current study seeks to increase the knowledge base of thermoplastic composites especially in terms of understanding the effect of temperature on the performance of PSU/GF composites. Additionally, the comparison of the present study results with the data obtained in [32] allows the effect of the polymer nature on the interaction between the matrix and GF in the composites to be revealed. 

## 2. Materials and Methods

### 2.1. Material and Sample Preparation

**Figure 1:**
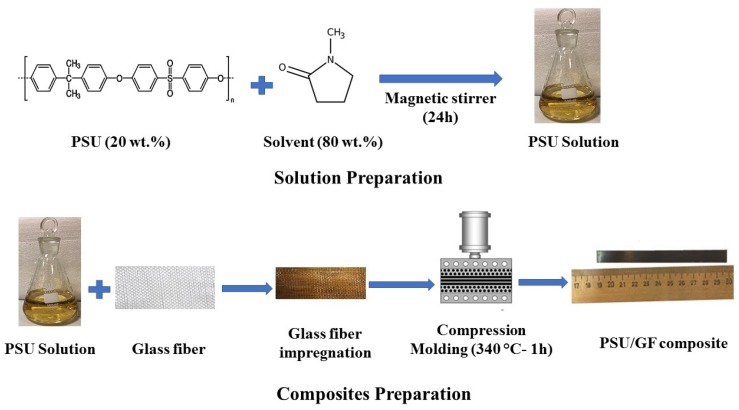
Scheme of the preparation of the polysulfone (PSU) solution and composites.

Woven glass fabrics (NPO “Stekloplastic”, Moscow, Russia) (T-23/1 “260 ± 10 g/m^2^) and PSU Ultrason S2010 (Basf, Ludwigshafen, Germany) powder were used as raw materials. A polysulfone solution was obtained by dissolving the PSU powder in N-methyl-2-pyrrolidone (Eastchem, Jiangsu, China). Bulk composite samples were formed in accordance with the method described in [32]. The solution was prepared in a 20/80 polymer to solvent weight ratio for 24 h using a magnetic stirrer. The samples were dried at a temperature of 150 °C for 5 h, and then they were compression molded at 340 °C and 10 MPa. Figure 1 shows a scheme of the preparation process of the PSU solution and composites. Three fiber to polymer weight ratios were prepared (50/50, 60/40, and 70/30 (wt.%)). There are many sizing compositions used in commercial GF, which can be completely wiped out by using a thermal treatment in a range of 200 to 400–500 °C [33,34,35,36,37,38]. The method of removing the sizing coating from the GF surface was elaborated previously [32]. Notably, the investigation was carried out using the same type of GF; it was shown that the optimal preheating conditions for the type of GF used is annealing in an air-atmosphere furnace at 350 °C for 1 h, according to Fourier-transform infrared (FTIR) spectra; preheated GF used in this study was prepared using the above-mentioned conditions. The composites were reinforced using initial and preheated GF.

### 2.2. Characterization of the Samples’ Structures

An FTIR spectrometer Nicolet 380 (Thermo Scientific, Waltham, MA, USA) (spectral range of 4000–450 cm^−1^, resolution of 1 cm^−1^) was used to study the chemical structures of the samples. The microstructure, fracture, interfacial bonding, and fiber pulling out were studied using a scanning electron microscope (VEGA 3 TESCAN) (TESCAN ORSAY HOLDING, a.s., Brno–Kohoutovice, Czech Republic) in backscattered electron image mode. Before the SEM examination, the samples were coated with a thin layer (10–15 nm) of carbon in a sputter coater.

### 2.3. Mechanical Tests

Flexural and shear properties were measured using a Zwick/Roell Z020 universal test machine (Zwick Roell Group, Ulm, Germany) provided with 1 and 20 kN sensors and a MultiXtens contact strain measurement system. Conforming with ISO 14125:1998 standards, the samples for the flexural tests were prepared in a dimension of 110 mm × 10 mm × 2 mm and 80 mm span. For shear tests (according to ASTM D 3846), 110 mm × 10 mm × 4 mm samples were used with a gauge length of 80 mm. According to this method, the shear strength was measured by applying a compressive load to a notched specimen of uniform width. The specimen was loaded edgewise in a supporting jig of the same description in ASTM D 695 for testing thin specimens. A failure of the specimen occurred in shear between two centrally located notches machined halfway through its thickness and spaced a fixed distance apart on opposing faces. The distance between the notches was 6.5–8 mm. The test speeds were 10 and 1.3 mm/min for the flexural and shear tests, respectively. At least five samples were examined at room temperature in each condition.

### 2.4. Thermo-Mechanical Tests

A DMA Q800 (TA Instruments, New Castle, DE, USA) dynamic mechanical analyzer was used to study the dynamic mechanical properties. The specimens sized 2 mm × 2 mm × 45 mm were used for the DMA tests. The measurements were realized using a double cantilever clamp at a frequency of 1 Hz and a deformation of 0.1%, in a temperature range from 30 to 220 °C; the heating rate was of 2 °C/min. The HDT tests were carried out using an Instron CEAST 6910 HDT/Vicat tester. The samples sized 80 mm × 10 mm × 4 mm were used in the HDT test at a load of 1.8 MPa and a span length of 64 mm (ISO 75). The deflection in the HDT test was set up to 1 mm as a maximum deflection. The DMA and HDT were performed for both PSU composites reinforced with initial and preheated GF. In each condition, three fiber to polymer weight ratios (50/50, 60/40, and 70/30 (wt.%)) were used.

## 3. Results and Discussion

### 3.1. FTIR

**Figure 2:**
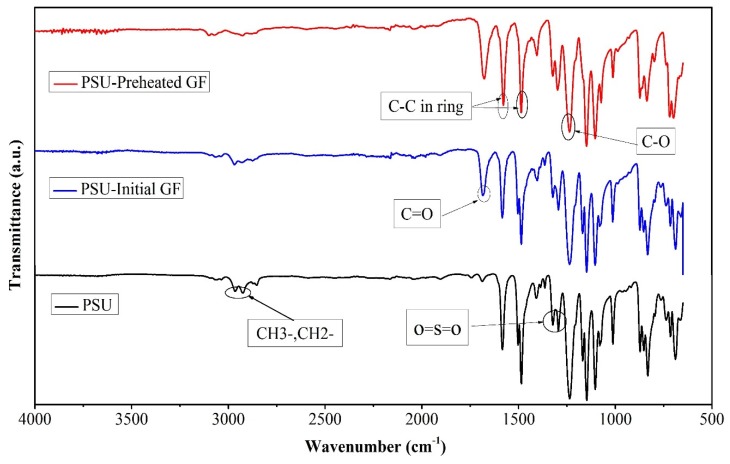
FTIR spectra for the initial PSU and 50/50 PSU composites reinforced with initial and preheated GF.

Figure 2 shows the FTIR spectra for the initial PSU and PSU reinforced with both initial and preheated GF 50/50 composites. For the initial PSU spectra, the C–H band for the aryl group was noticed in a range of 3000–3100 cm^−1^. The peaks at 2800 and 3000 cm^−1^ related to symmetric and asymmetric bands of CH_3_ and CH_2_. The C–C in-ring bands were revealed by 1401, 1501, and 1586 cm^−1^ peaks. The stretching vibration of the asymmetric O=S=O band occurred at 1292 and 1325 cm^−1^, while the peak at 1232 cm^−1^ referred to the stretching vibration of the C–O band. The stretching of the symmetric O=S=O bands appeared at 1140 and 1168 cm^−1^, and the aryl group was indicated by 1019 cm^−1^ peak [1,23,39,40].

Few differences could be distinguished in the spectral attribution of PSU composites. In the spectra of the composites, the C=O band appeared clearly due to the presence of some residual solvent [32]. The amplitude of this peak was stronger in the spectra related to the PSU reinforced with preheated GF due to the oxidation during the preheating process. In the case of the composites containing preheated GF, the peaks between 2800 and 3000 cm^−1^ reduced because of the removal of GF sizing [32]. Based on the data observed from FTIR, it could be noted that the spectrum characteristic for the composite was very similar with that of PSU except some new peaks because of the presence of some residual solvent and the effect of the removal of GF coating.

### 3.2. Mechanical Tests

**Figure 3:**
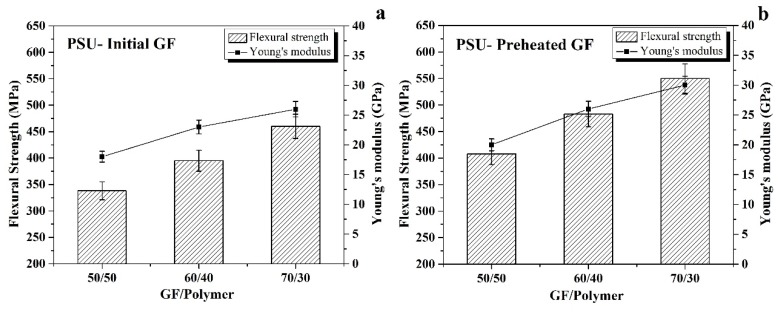
Flexural strength and Young’s modulus for the initial GF (**a**,**b**) the preheated GF reinforced composites.

The flexural and shear tests were implemented to study the mechanical properties of the composites. The comparison of flexural strength and Young’s modulus values for the initial GF reinforced composites are shown in Figure 3a. The curve showed a trend of increasing flexural strength and Young’s modulus with increasing the GF ratio. The composites with a GF to PSU ratio of 70/30 recorded the maximum value of flexural strength (460 MPa) and Young’s modulus (26 GPa) compared with the 340 MPa and 18 GPa for the 50/50 composites. The comparison of these data with those observed previously for polyethersulfone based composites [32] shows that in case of PSU matrix composites, no decrease in Young’s modulus at the increase of the GF content from 60/40 to 70/30 was observed.

The sizing coating prevents good adhesion between the fiber and the polymer, which mainly affects the composite’s strength. A thermal treatment was carried out to remove the sizing coating of the fiber to enhance the interface bonding between the polymer and the fibers [32,41]. Figure 3b illustrates the values of flexural strength and Young’s modulus for the preheated GF reinforced composites. It can be noted that the mechanical properties increased with increasing the GF content; the flexural strength increased from 408 MPa for 50/50 composites and 483 MPa for the 60/40 composites to 550 MPa in the case of the 70/30 composites, whereas Young’s modulus increased from 20 GPa for the 50/50 composites to 26 and 30 GPa for the 60/40 and 70/30 composites, respectively. A remarkable enhancement occurred in the preheated GF composite properties compared with the initial GF composites at the same ratio. It was considered that heating the GF removed the GF sizing, which contributed to the improvement of interface bonding between the fiber and the matrix. 

**Figure 4:**
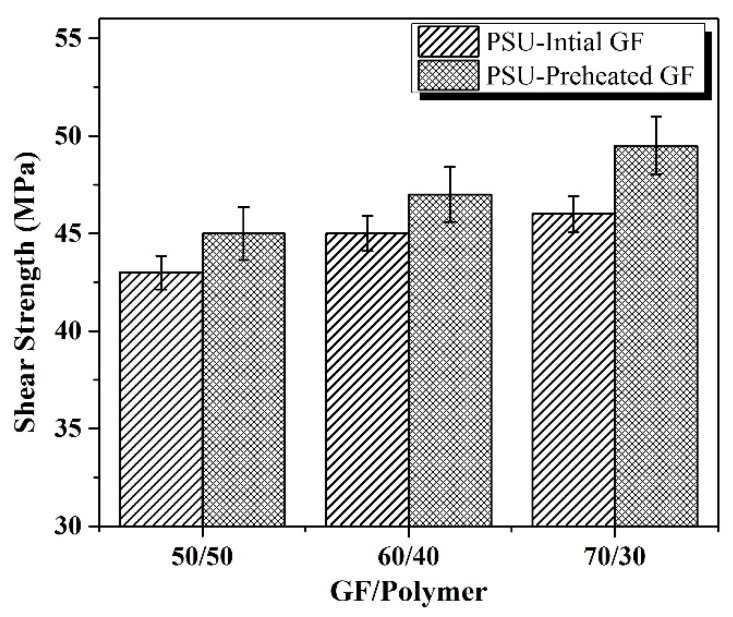
Shear strength for the initial GF and the preheated GF reinforced composites.

Shear strength, which is affected mainly by the interface bonding, is illustrated in Figure 4. The effect of the removal of the GF sizing on the interface between GF and the matrix was clearly demonstrated by an increase of shear strength values of the preheated GF composites compared with those of the initial GF composites at the same ratios shown in the figure. Shear strength increased from 43 MPa for the initial GF to 45 MPa for the preheated GF 50/50 composites and from 45/46 MPa for the initial GF 60/40 and the 70/30 composites to 47/49.5 MPa for the preheated GF 60/40 and 70/30 composites, respectively. The comparison of these data with those observed previously for polyethersulfone based composites [32] shows that in case of the PSU matrix composites, shear strength tended to increase with an increase in the GF content from 60/40 to 70/30 both for the composites containing initial and preheated GF, whereas for polyethersulfone based composites such an increase in the GF content resulted in a decrease in shear strength even for the composites reinforced with the preheated GF. It is additional evidence of the important role of the chemical nature of the matrix polymer on the interaction between the matrix and the reinforcers. 

### 3.3. Thermo-Mechanical Tests

**Figure 5:**
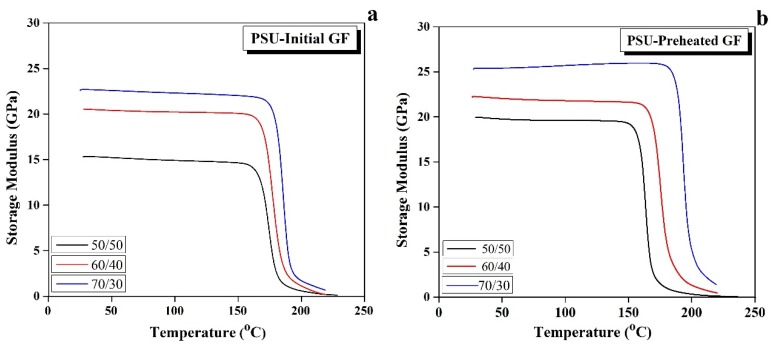
Temperature dependences of the storage modulus for the initial (**a**,**b**) the preheated GF reinforced composites.

Figure 5a shows the temperature dependences of the storage modulus for the initial GF reinforced composites. The values of the storage modulus remained at a plateau in a temperature range below the *T*_g_, while it started to fall around the *T*_g_, which is the region of the transformation from glassy to rubbery state. It can be noted that the storage modulus increased with an increase in the GF content as a result of an increase in the stiffness and the thermo-resistance of the composites with increases in the GF ratio. The results recorded that the storage modulus of 22 GPa was found for the 70/30 composites, while the storage modulus values for the 50/50 and 60/40 composites were 15.5 and 20.5 GPa, respectively. It is considered that the thermo-mechanical characteristic of the composite was improved with increasing the GF/PSU ratio due to the enhancement of thermal stability of the composite as a result of an increase of the composite’s stiffness and the interfacial interaction, which increased the thermodynamic compatibility between GF and the polymer [21,25]. 

The effect of using the preheated GF on the storage modulus of the composites is investigated in Figure 5b. The data showed a noticeable enhancement of the storage modulus values compared with the initial GF composites at the same ratio. This increase was attributable to the improvement of the stiffness of the preheated GF composites due to an increase of the interface between the fiber and the polymer after removing the GF coating, which affected directly on the storage modulus and the ability of the material to store energy. The storage moduli of the 50/50, 60/40, and 70/30 preheated GF composites were 20, 22, and 26 GPa, respectively. 

**Figure 6:**
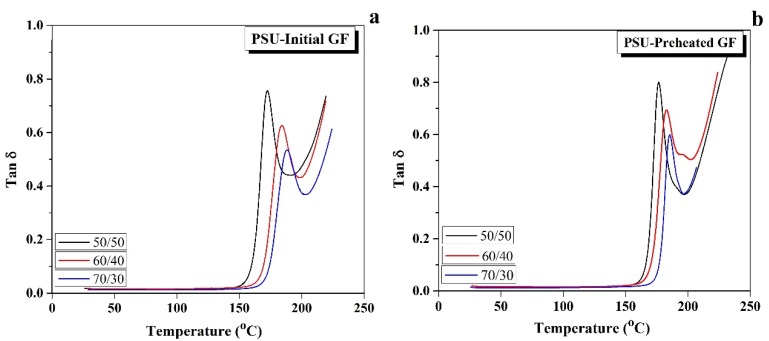
Temperature dependences of Tan δ for the initial (**a**,**b**) the preheated GF reinforced composites.

Another way to evaluate thermal stability of the composites’ mechanical properties is tangent delta (tan δ) measurement. Tan δ refers to the ratio between loss and storage modulus, and the peak on the Tan δ curve refers to the *T*_g_, which differentiates between the glassy and rubbery region of the thermo-mechanical behavior of the composite. Tan δ of a different initial GF to polymer ratio is shown in Figure 6a. It can be seen that the tan δ maximum decreased with an increase in the GF to PSU ratio, i.e., it decreased from 0.75 for 50/50 composites to 0.65 and 0.53 for 60/40 and 70/30 composites, respectively. On the other hand, the *T*_g_ increased from 163 °C for the 50/50 composites to 180 and 192 °C for the 60/40 and 70/30 composites, respectively, due to an increase of thermal stability of the composites. The reduction behavior of tan δ was due to a decrease of the molecular chain’s mobility as a result of increasing the fiber/polymer interface bonding [2]. With using the preheated GF, the composites became stiffer so that the values of tan δ decreased in the preheated composites, as shown in Figure 6b, compared with the same ratio of the initial GF reinforced composites. The improvement achieved from using the preheated GF raised thermal stability, which in turn enhanced the *T*_g_ of the composites. The results showed an increase from 170 °C for the 50/50 composites to 187 and 198 °C for the 60/40 and 70/30 composites, respectively.

**Figure 7:**
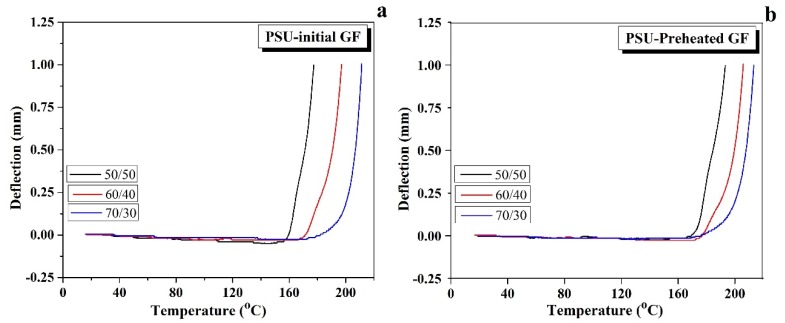
Heat deflection curves for (**a**) the initial and (**b**) the preheated GF reinforced composites.

The HDT tests for the initial and preheated GF reinforced composites were carried out to study the deformation behavior of the composites at the evaluated temperature. The maximum deflection was set to be 1 mm. The HDT results of the initial GF reinforced composites, shown in Figure 7a, indicated that the deflection remained approximately zero up to a temperature near the *T*_g_ of the polymer matrix and drastically increased above the *T*_g_. The HDT for the initial GF reinforced composites enhanced from 168 °C for the 50/50 composites to 197 and 209 °C for the 60/40 and 70/30 composites, respectively. It can be proposed that the HDT increases as a result of stiffness and thermal stability enhancement with increasing the GF/PSU ratio [30,42]. The value of deflection works as an indicator of thermal stability. As it is shown in Figure 7, the deflection of the composites was near to zero upon the *T*_g_, whereas above the *T*_g_ the deflection increased rapidly. The same behavior was observed in the case of the preheated GF composites, as seen in Figure 7b. The HDT for the composites containing the preheated GF were 181, 202, and 214 °C for the 50/50, 60/40, and 70/30 composites, respectively. The HDT values were found to be higher in the preheated GF composites than those in the initial GF composites. This can be explained by an increase in thermal stability of the composite along the improvement in the interface bonding between the fiber and the matrix.

The values of the *T*_g_, tan δ, and HDT for the initial and preheated GF composites are given in Table 1. It can be noticed that the thermal properties improved with use of the preheated GF instead of the initial GF in polysulfone composites.

**Figure 8:**
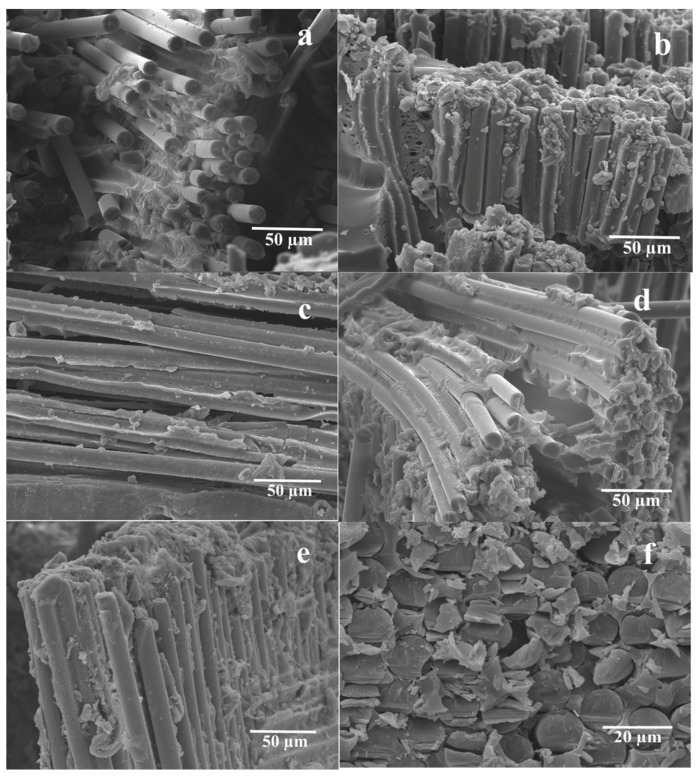
SEM images of the flexural fracture surfaces of the 50/50 (**a**,**b**), 60/40 (**c**,**d**), and 70/30 (**e**,**f**) PSU based composites reinforced with the initial (**a**,**c**,**e**) and the preheated (**b**,**d**,**f**) GF.

Figure 8 shows the microstructure of the flexural fracture surface of the PSU based composites reinforced with the initial and preheated GF. Pull-out phenomena appeared in the case of the 50/50 initial fiber-reinforced composites (Figure 8a), which means that the adhesion on the PSU/GF interface, in this case, was not sufficient. Preheating resulted in the improvement in the boundary adhesion (Figure 8b), which was confirmed by the formation of a large amount of PSU particles adherent to the fiber surface. Some pores appeared in the 50/50 preheated fiber-reinforced composites (Figure 8b) because of the presence of some solvent that was not removed during the drying process before compression molding. The evaporation of the solvent resulted in the formation of pores during compression molding. The preheated fiber-reinforced composites of 60/40, as seen in Figure 8d, also showed better interface bonding between the fiber and the polymer than those in the 60/40 initial GF composite shown in Figure 8c. Good interface bonding between the fiber and the matrix occurred in the 70/30 initial GF reinforced composites, as shown in Figure 8e. As seen in Figure 8f, the 70/30 preheated GF reinforced composites, the fracture of fiber was in a brittle form, which was due to sufficient interface bonding between the fiber and the polymer. Moreover, the distribution of the polymer was improved. Thus, an increase in the interfacial interaction due to GF preheating resulted in higher mechanical properties of the composites.

## 4. Conclusions

Mechanical and thermo-mechanical properties of the PSU composites reinforced with initial and preheated GF for a different fiber to polymer weight ratio were studied. The flexural test showed that the composite stiffness and Young’s modulus enhanced with increasing the fiber ratio in the initial GF reinforced composites. A remarkable improvement was achieved by using a preheated GF to reinforce PSU. Additionally, shear strength increased in the cases of using a preheated GF. The storage modulus, tangent delta, and *T*_g_ values obtained from the DMA test and HDT obtained from the HDT test were increased in the preheated reinforced GF composites compared with those in the initial GF reinforced composites. The fiber to polymer ratio of 70/30 recorded the best properties for the initial and preheated GF reinforced composites. The 70/30 initial GF composites recorded 460 MPa, 26 GPa, and 22 GPa for flexural strength, Young’s modulus, and storage modulus, respectively. Due to the improvement of the interfacial adhesion, these magnitudes were increased in the case of the 70/30 preheated GF composites to record 550 MPa, 30 GPa, and 26 GPa for flexural strength, Young’s modulus, and storage modulus, respectively. FTIR of the PSU composites showed the main peaks of PSU and GF for the initial and preheated composites. Additionally, the FTIR spectra showed that the sizing coating was removed by heating the GF. It revealed that some of the solvent was not disposed of during the drying process. The SEM images showed a good distribution of the polymer on the GF surface, which improved with using the preheated GF that led to an increase in the interface bonding between the polymer and GF. 

## Figures and Tables

**Table 1:** The *T*_g_, tan δ, and HDT values for the composites reinforced with the initial and preheated GF.

Fiber/Polymer	50/50			60/40			70/30		
Property	*T*_g_ (°C)	Tan δ	HDT (°C)	*T*_g_ (°C)	Tan δ	HDT (°C)	*T*_g_ (°C)	Tan δ	HDT (°C)
Initial GF composites	163	0.75	168	180	0.65	197	192	0.53	209
Preheated GF composites	170	0.81	181	187	0.7	202	198	0.56	214
